# Plant Extracts in Obesity: A Role of Gut Microbiota

**DOI:** 10.3389/fnut.2021.727951

**Published:** 2021-09-23

**Authors:** Guangying Weng, Yehui Duan, Yinzhao Zhong, Bo Song, Jie Zheng, Shiyu Zhang, Yulong Yin, Jinping Deng

**Affiliations:** ^1^Guangdong Provincial Key Laboratory of Animal Nutrition Regulation, South China Agricultural University, Guangzhou, China; ^2^CAS Key Laboratory of Agro-Ecological Processes in Subtropical Region, Hunan Provincial Key Laboratory of Animal Nutritional Physiology and Metabolic Process, National Engineering Laboratory for Pollution Control and Waste Utilization in Livestock and Poultry Production, Institute of Subtropical Agriculture, Chinese Academy of Sciences, Changsha, China; ^3^College of Advanced Agricultural Sciences, University of Chinese Academy of Sciences, Beijing, China

**Keywords:** plant extracts, obesity, gut microbiota, mechanisms, lipid metabolism

## Abstract

Obesity has become one of the most serious chronic diseases threatening human health. Its occurrence and development are closely associated with gut microbiota since the disorders of gut microbiota can promote endotoxin production and induce inflammatory response. Recently, numerous plant extracts have been proven to mitigate lipid dysmetabolism and obesity syndrome by regulating the abundance and composition of gut microbiota. In this review, we summarize the potential roles of different plant extracts including mulberry leaf extract, policosanol, cortex moutan, green tea, honokiol, and capsaicin in regulating obesity via gut microbiota. Based on the current findings, plant extracts may be promising agents for the prevention and treatment of obesity and its related metabolic diseases, and the mechanisms might be associated with gut microbiota.

## Introduction

In recent years, obesity has been increasing at an alarming rate worldwide and seriously affects not only developed countries but global people (1). Obesity is characterized by a series of metabolic disorders, especially lipid dysmetabolism and its complication (2). Lipid metabolism involves the biosynthesis and degradation of lipids such as triglycerides, cholesterol, phospholipids, and fatty acids (3). The disorder of lipid synthesis and decomposition processes can lead to lipid metabolism dysregulation (also known as lipid dysmetabolism), subsequently giving rise to the progression of obesity and its related metabolic diseases such as diabetes (4–7). Thus, maintaining the balance of lipid metabolism is of great importance to prevent and treat obesity.

Obesity is regulated by various factors such as genetic factors, dietary habits (e.g., high glycaemic diets), underlying diseases (e.g., insulinoma), and exercise (8). Recently, direct evidence pointed to a strong relationship between obesity and gut microbiota (9). The colonization of germ-free mice with a “normal microbiota” from conventionally raised mice resulted in an increase in body fat mass despite reduced food intake (10). Similarly, germ-free mice fed low-fat chow were colonized with obese co-twin's fecal microbiota presenting obese phenotype compare with those colonized with lean co-twins' fecal microbiota (11). Further evidence comes from the finding that total fecal microbiota transplantation from normal mice significantly attenuated high-fat diets-induced lipid dysmetabolism in mice (12). Moreover, obese individuals exhibited significant alteration in gut microbiota compared with lean controls, as manifested by increased ratio of *Firmicutes* to*Bacteroidetes* (13, 14). In addition, increasing lines of evidence suggested that obesity and its related metabolic diseases exert seriously adverse effects on the structure of host gut microbiota, as indicated by a significant alteration in gut microbiota composition and diversity (11, 12, 15–17). In turn, gut microbiota dysregulation may not only increase the intestinal permeability to gut microbes but also elevate the production of harmful microbial metabolites, thus aggravating lipid dysmetabolism and resulting in obesity and its related diseases, such as diabetes and nonalcoholic fatty liver disease (NAFLD) (18, 19). Accordingly, gut microbiota plays important role in the regulation of obesity and lipid metabolism, and it may be a potential therapeutic target for ameliorating obesity.

**Figure 1:**
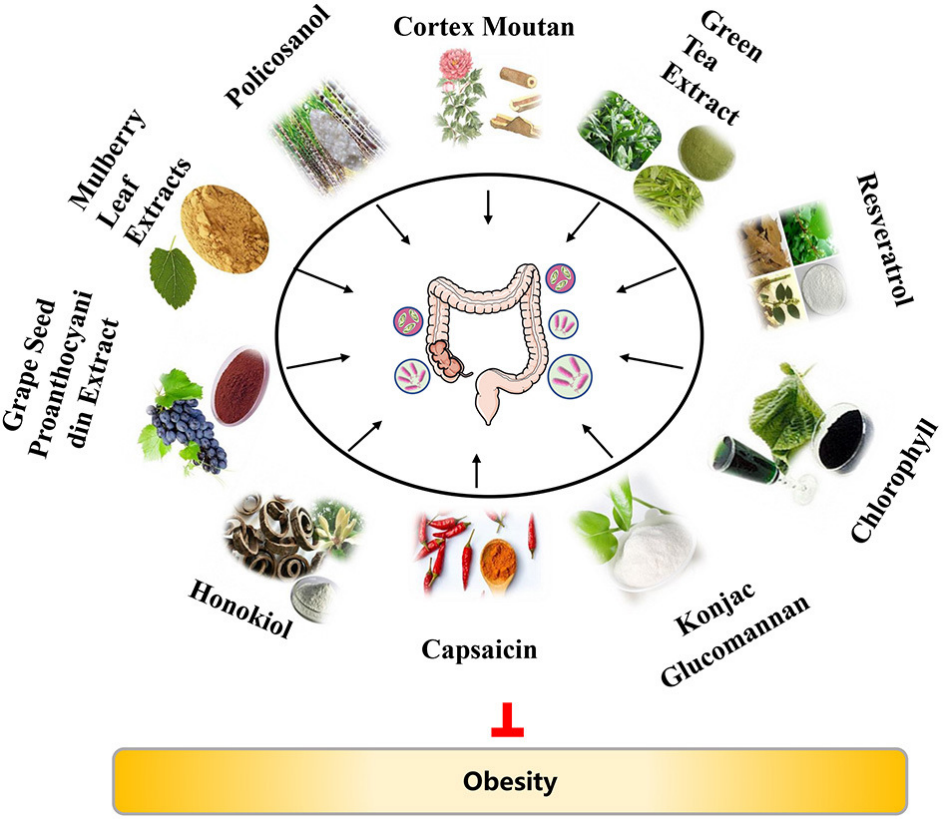
The overview of anti-obesity effects of plant extracts via gut microbiota.

Over the past years, increasing evidence has demonstrated that diets and/or nutrients exert roles in the regulation of gut microbiota composition and obesity (3, 7, 20–22). In this review, we will discuss the anti-obesity activity of plant extracts, with highlighting of their potential mechanisms related to gut microbiota, in order to provide an updated understanding of the relationship among plants extracts, gut microbiota, and obesity (Figure 1). We hope that this review can provide some available information to develop dietary strategies for the treatment and prevention of obesity and its related diseases.

## Plant Extracts in Obesity: Potential Implication of the Gut Microbiota

### Mulberry Leaf Extracts

**Figure 2:**
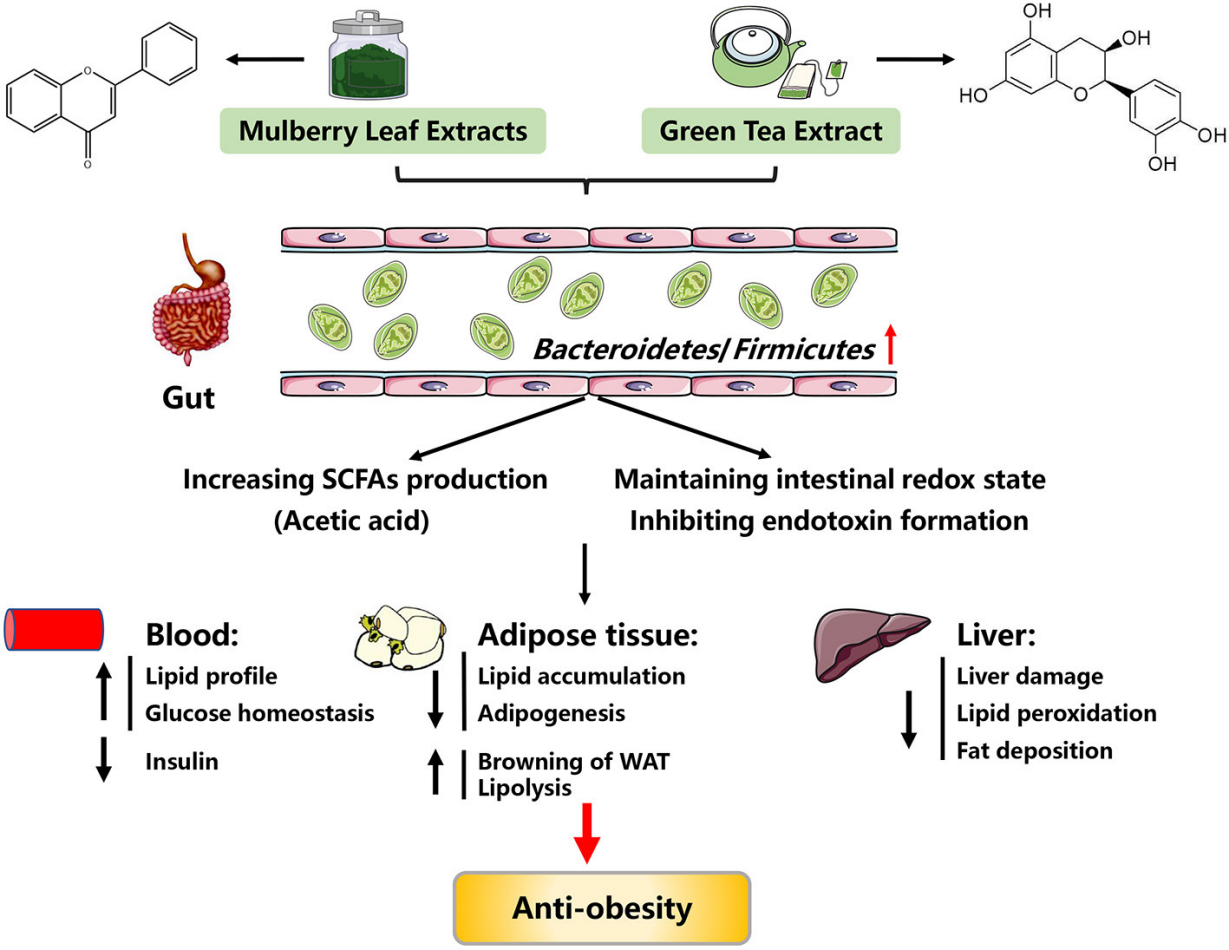
Possible mechanisms explaining the anti-obesity effects of mulberry leaf extracts and green tea extracts. SCFAs, short chain fatty acids; WAT, white adipose tissue.

Flavonoids from mulberry leaves (FML) are one of the main functional components of mulberry leaf extracts, which are edible food and widely used as a kind of traditional Chinese medicine. It has been reported that mulberry leaf extracts such as FML possess multiple biological activities, such as antioxidant, improving skeletal muscle function, cardioprotective, and anti-cancer (23–26). In addition, there is evidence showing that mulberry leaf extracts confer anti-obesity effects (Figure 2).

Evidence from hyperlipidemic mice showed that FML treatment (30 mg kg^−1^ body weight) for 12 h improved blood lipid metabolism, as evidenced by reduced levels of serum total triglycerides (TG), total cholesterol (TC) and low-density lipoprotein cholesterol (LDL-C) by 152, 207, and 110 mg/100 mL, respectively (27). Similar improvements in blood lipid metabolism were also observed in high fat diets (HFD)-fed mice, in which FML administration (240 mg kg^−1^ body weight) for 6 weeks could also reduce body weight gain and adipose tissue mass, and alleviate the whitening of brown adipose tissue (BAT) (28). Moreover, mulberry water extracts could reduce lipid peroxidation and lipid accumulation in the liver of rats (29). Apart from using alone, mulberry leaf extracts combined with mulberry fruit extract also exhibited anti-obesity effects in HFD-induced obese mice, as evidenced by decreased body weight gain, improved fasting plasma glucose and insulin, and alleviated inflammation and oxidative stress (30). Consistent with *in vivo* studies, *in vitro* studies have also reported that treatment of mulberry leaf extracts to aortic vascular smooth muscle cells (VSMCs) could inhibit its proliferation and migration, thus preventing atherosclerosis, a disease related to lipid dysmetabolism (31).

Although much of the work in this field has been done on rodent models, these models have yielded important insights into the anti-obesity mechanisms of mulberry leaf extracts. Since flavonoids are enzymatically hydrolyzed by gut microbiota and absorbed in the intestine (32), it is posited that the beneficial effects of FML might be *via* gut microbiota. In support, FML treated-obese mice exhibited increased *Bacteroidetes* abundance and decreased *Firmicutes* abundance (28, 33, 34). Increased *Bacteroidetes* abundance led to an elevation in the production of acetic acid, which further promotes lipolysis and inhibits lipid accumulation through activating G protein-coupled receptor 43 (GPR43), a short chain fatty acid receptor (28, 35). Taken together, the anti-obesity mechanisms of mulberry leaf extracts are summarized in Figure 2.

### Policosanol

Policosanol is a natural mixture of long-chain aliphatic primary alcohols extracted from the wax constituent of plants and seeds, such as sugar cane, wheat, corn, sesame, soybean, perilla seed, grape seed and rice bran, whose main ingredient is octacosanol (36). Policosanol has multiple bioactivities, such as anti-nociceptive, anti-inflammatory, anti-cancer, and anti-parkinsonian properties (37–41). In addition, policosanol also exerts beneficial effects on lipid dysmetabolism and obesity, such as improving blood lipid profile, elevating BAT activity, improving glucose homeostasis, and regulating cholesterol synthesis (42–44).

First, evidence from human subjects has shown that the reduction of serum high-density lipoprotein-cholesterol (HDL-C) levels and the elevation of serum TG, TC and glucose levels were reversed by policosanol treatment (10 mg day^−1^) for 8 weeks, indicative of an anti-obesity effect (42, 45). Similar reduction of serum TG levels was also obtained in HFD-fed rats, in which octacosanol treatment (10 g kg^−1^ diet) for 20 days also significantly reduced perirenal adipose tissue weight (46). Apart from the regulation of blood lipid profile and adipose tissue mass, policosanol treatment (60 mg kg^−1^ day^−1^, for 4 weeks) could attenuate insulin resistance and improve glucose homeostasis by elevating BAT activity and reducing hepatic lipid content in HFD-induced obese mice (43). In addition, policosanol also exerts roles in regulating cholesterol metabolism. For example, policosanol treatment (0.38–1.5 g kg^−1^ diets) in hamsters greatly decreased serum levels of total cholesterol by 15–25% and increased the excretion of acidic sterols (the cholesterol end-product) by 25–73%, indicating that policosanol lowered cholesterol via restraining the absorption of bile acids (44). One potential mechanism for the hypocholesterolemic effect of policosanol is its down-regulation of 3-hydroxy-3-methylglutary coenzyme A (HMG-CoA) reductase, the key rate-limiting enzyme in cholesterol biosynthesis (47, 48). In support, an *in vitro* study found that policosanol suppressed HMG-CoA reductase activity by activating adenosine 5′-monophosphate-activated protein kinase (AMPK) (48, 49).

Cholesterol degrading into bile acids functions as the main way to expel excess cholesterol from host, which can effectively reduce the risk of atherosclerosis (50). On the other hand, bile acids act as an important regulator of cholesterol metabolism. The biosynthesis and biotransformation of bile acids are closely associated with host gut microbiota. In detail, primary bile acids are synthesized and secreted by host hepatocytes, and then transformed into secondary bile acids with the chemical modification effect of gut microbiota (51). The disorder of gut microbiota results in bile acid dysmetabolism and in turn affects cholesterol metabolism, suggesting that the regulation effects of policosanol on cholesterol metabolism is partially mediated by gut microbiota (52–55). In summary, these findings show that policosanol seems to be a promising phytochemical alternative to classic cholesterol-lowing agents such as statins. However, the detailed mechanisms of the mode of policosanol's action remain unclear, but alterations in gut microbiota are probably involved.

### Cortex Moutan

**Figure 3:**
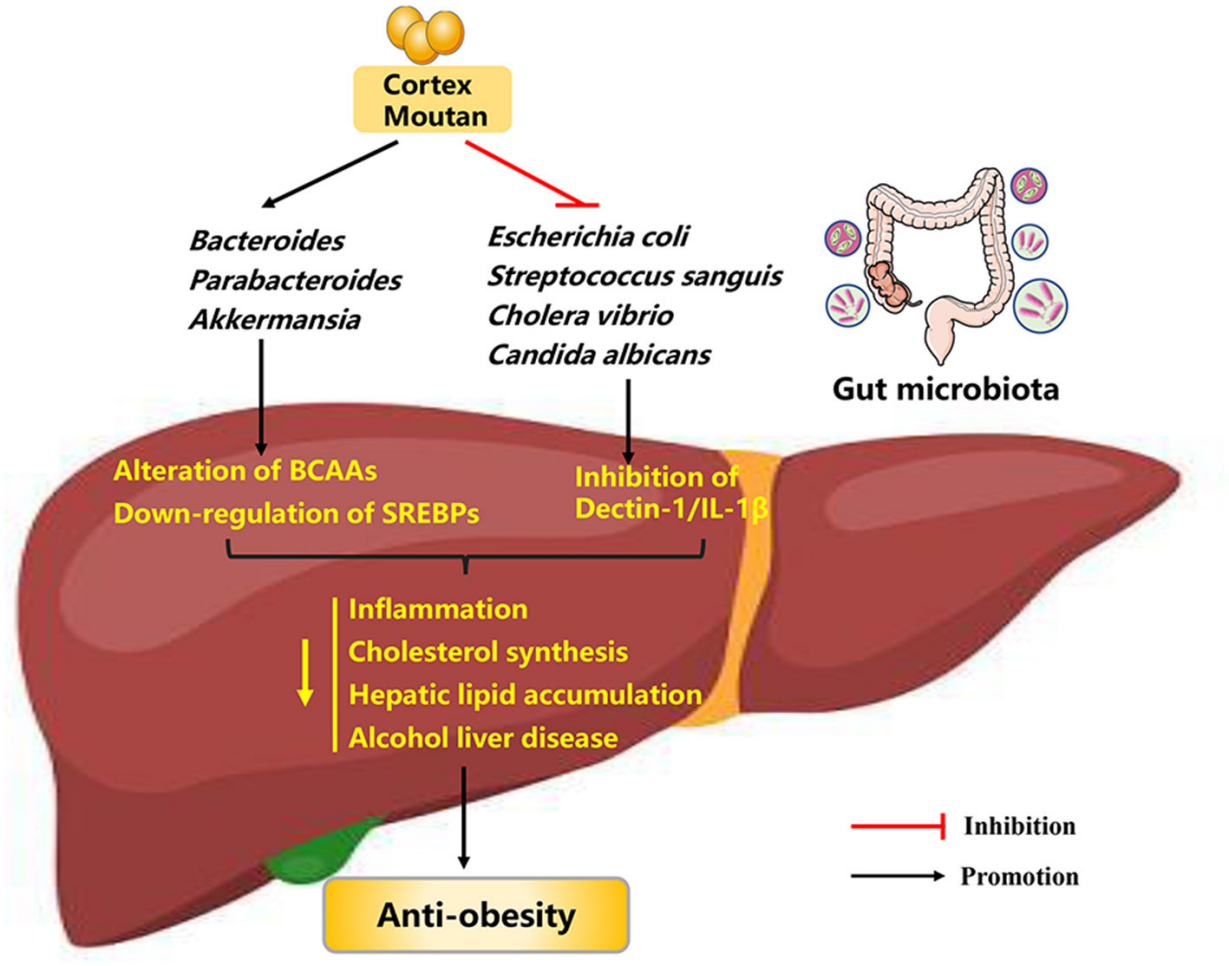
Possible mechanisms explaining the anti-obesity effects of cortex moutan. BCAAs, branched-chain amino acids; SREBPs, sterol-regulatory element binding proteins; IL-1β, interleukin-1β.

Cortex Moutan (CM), the root bark of *Paeonia suffruticosa* Andrews, is widely used as a traditional Chinese herbal medicine that has multiple bioactive ingredients. Both CM and its bioactive ingredients possess many pharmacological properties on cardiovascular diseases, anti-tumor, and nervous system (56–58). The main pharmacological effects of CM are attributed to its bioactive component paeonol. Recently, more and more studies have found that CM and paeonol are also able to regulate preadipocyte differentiation, glucose homeostasis, lipid peroxidation, and inflammatory response, thus mitigating obesity (Figure 3).

It has been reported that administration of paeonol (150 and 300 mg kg^−1^) to diabetic mice for 8 weeks resulted in a reduction in fasting blood glucose, serum TG and TC, hepatic TG and TC though activation of serine/threonine kinase B (Akt) (59). Similar improvements in lipid metabolism were obtained in myocardial ischemia rabbits (60). In addition, paeonol administration could ameliorate lipid peroxidation and inflammatory response (61, 62). In HFD-induced atherosclerotic rabbits, paeonol exerts anti-atherosclerosis effects by inhibition of lipid peroxidation and improvement of anti-inflammatory action (63). In accord with these findings in *in vivo* studies, several *in vitro* studies have reported that paeonol can markedly and dose-dependently reduce intracellular lipid accumulation in mouse preadipocytes and macrophages, and delay preadipocytes differentiation into mature adipocytes, thus protecting against metabolic diseases (64, 65). Overall, overwhelming evidence suggests that paeonol treatment can combat obesity and its related metabolic diseases such as atherosclerosis and steatohepatitis.

Since its low bioavailability and phenolic hydroxyl groups in its chemical structure, CM can interact with gut microbiota in the intestinal tract over extended periods of time (66, 67). These observations raise the possibility that gut microbiota may play a role in the mode of CM's action (Figure 3). Evidence to support this hypothesis is that CM possess antimicrobial activity against a broad range of bacteria, such as *Escherichia coli, Streptococcus sanguis*, and *Cholera vibrio* (68, 69). Further evidence for a relationship between CM treatment and gut microbiota comes from the findings that CM administration (0.4 g crude drug kg^−1^, either along or as part of a therapeutic regimen) to HFD-induced obese mice for 6 weeks significantly reverse the composition and diversity of gut microbiota, as evidenced by restored abundances of *Bacteroides, Parabacteroides, Akkermansia*, and *Mucispirillum*. Subsequently, improved gut microbiota could affect the levels of blood metabolites such as branched-chain amino acids (BCAAs), and down-regulate the expression of sterol-regulatory element binding proteins (SREBPs, crucial regulators controlling cholesterol and fatty acid *de novo* synthesis) in the liver, thus alleviating obesity (70). In addition, paeonol can reduce intestinal fungal abundance (especially *Candida albicans* abundance) and inhibit the mycobiota-mediated dectin-1/interleukin-1β (IL-1β) signaling pathway, thus ameliorating alcohol liver disease in mice (71). In summary, CM and paeonol exert anti-obesity effects through the gut microbiota-blood metabolites-liver axis and microbiota-mediated signaling pathway, making them potential pharmaceutical agents against obesity.

### Green Tea Extract

Green tea, the unfermented dried leaves of *Camellia sinensis*, is one of the most popularly traditional beverages worldwide. The major bioactive ingredients of green tea are flavan-3-ols (also known as catechins), which are natural plant-derived and powerful antioxidant for alleviating oxidative stress. In particular, (–)-epigallocatechin-3-gallate (EGCG) is the most abundant and active catechin in green tea. Apart from antioxidant, green tea also possesses anti-obesity and anti-diabetic effects (72, 73).

*In vitro* studies presented evidence showing that catechin-rich green tea extract (GTE) could ameliorate lipid accumulation through inhibiting the differentiation of 3T3-L1 preadipocytes into adipocytes and stimulating the browning of white adipocytes (72, 74, 75). Similarly, in 3T3-L1 adipocytes, EGCG could improve glucose homeostasis through normalizing the redox imbalance and mitochondrial dysfunction (75). In line with these findings, *in vivo* studies also reported a beneficial role of GTE on obesity. For instance, evidence from human studies showed that daily ingestion of GTE (containing 583 mg of catechins) for 12 weeks resulted in decreases in body weight, adipose tissue mass, and serum LDL-C levels (76, 77). Similar observations were also obtained in diets-induced obese rodents, in which GTE was supplemented at a dose of 500 mg kg^−1^ body weight for 12 weeks, and the mechanisms might be related to AMPK activation and down-regulated microRNA 335 expression in the adipose tissue (78, 79). Apart from the above-mentioned roles, GTE supplementation could mitigate inflammation, enhance energy expenditure, and attenuate insulin resistance (80, 81). Altogether, GTE is a beneficial food constituent for preventing and treating obesity.

A potential mechanism for the anti-obesity of GTE was attributed to the alteration of gut microbiota, based on observations of increased *Bacteroides* abundance in response to GTE treatment (82–84). Two mechanisms may be responsible for the beneficial effects of GTE on obesity via improving gut microbiota. One hypothesized mechanism may be the increased production of short chain fatty acids (SCFAs) and the inhibition of endotoxin formation and translocation, thus attenuating obesity-associated adipose inflammation and decreasing body weight (84, 85). Another hypothesized mechanism may be via gut microbiota-improved intestinal redox state (86). Despite these positive outcomes, there is evidence showing that treatment of obese mice with overdose tea polyphenols would present side effects on their intestinal health (86). Taken together, GTE can exert protective roles against obesity, and its underlying mechanisms might be associated with gut microbiota (Figure 2). In future, more efforts should be made to investigate how GTE targets the specific gut microbiota.

### Resveratrol

**Figure 4:**
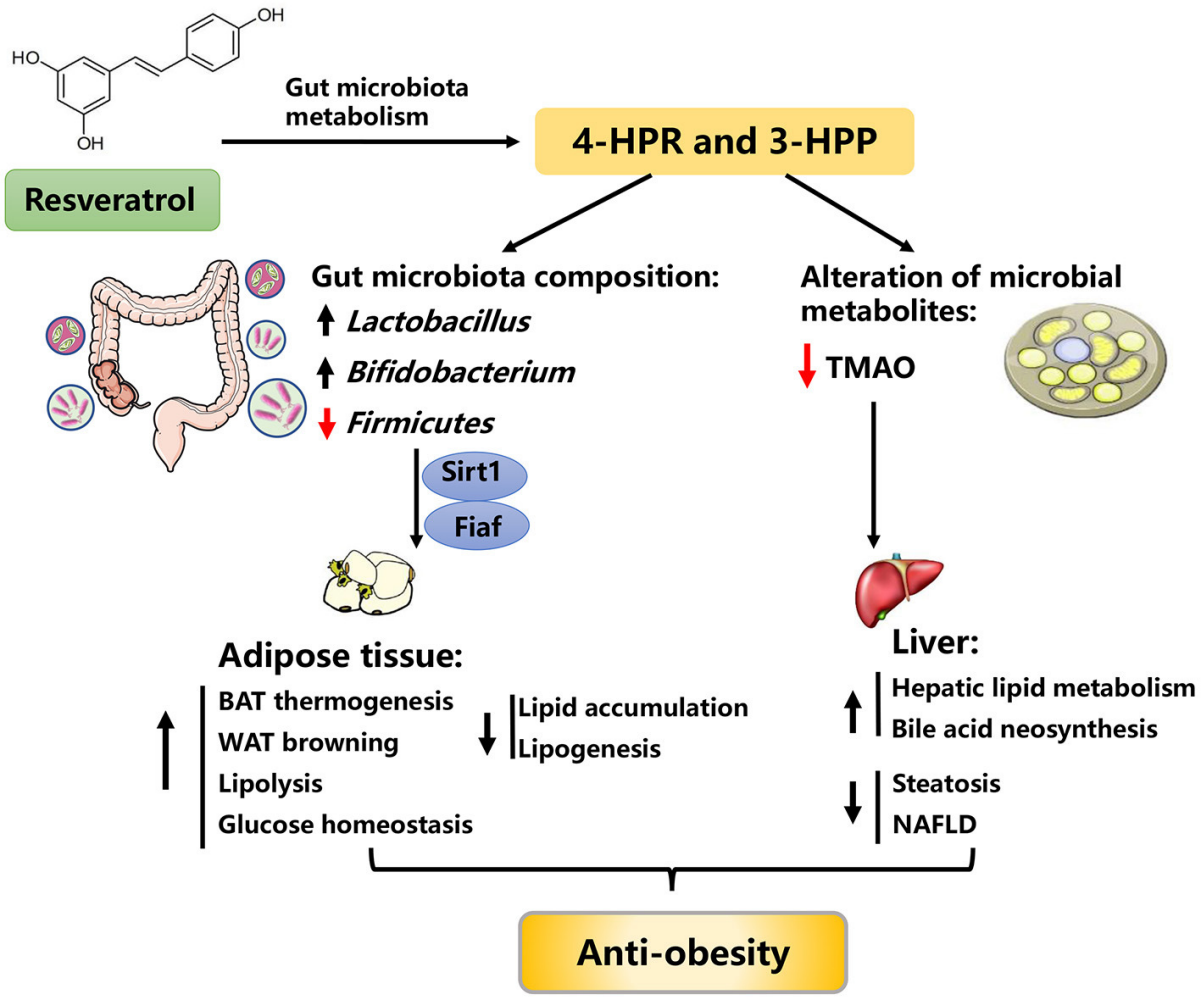
Possible mechanisms explaining the anti-obesity effects of resveratrol. 4-HPA, 4-hydroxyphenylacetic acid; 3-HPP, 3-hydroxyphenylpropionic acid; Fiaf, fasting-induced adipose factor; Sirt1, sirtuin 1; TMAO, trimethylamine-N-oxide, BAT, brown adipose tissue, NAFLD, nonalcoholic fatty liver disease.

Resveratrol (3, 5, 4′-trihydroxystilbene, RES)is a plant-derived polyphenolic compound, which could be isolated and purified from a variety of plants, such as grape, peanut, mulberry and polygonum cuspidatum. Over the past decade, RES is also one of the most studied plant active ingredients, because of its presumed pharmacological effects on cancer, cardiovascular diseases, and alzheimer's diseases (87–90). Meanwhile, it has been reported that RES also plays a crucial role in mitigating obesity, as evidenced by increased energy expenditure, BAT activity, white adipose tissue (WAT) browning, and glucose homeostasis (Figure 4).

In obesogenic diets-fed rats, RES supplementation (30 mg/kg/day, for 6 weeks) increased thermogenesis in skeletal muscle and BAT by upregulating uncoupling protein (UCP) expression, consequently improving whole-body energy dissipation and attenuating obesity (91). Likewise, supplementation of 4% RES to db/db mice for 10 weeks could enhance BAT activity and WAT browning and improve glucose homeostasis (92). Daily delivery of 300 mg kg^−1^ RES to HFD-fed mice for 16 weeks improved hepatic lipid metabolism and reduced liver steatosis, thus alleviating NAFLD (93). Administration of RES (10 mg/kg) to atherosclerotic mice for 24 weeks significantly reduced the intestinal fatty acid and monoglyceride accumulation, conferring beneficial effects on cardiovascular health (94). Consistent with these findings, several *in vitro* studies using stromal vascular cell model also demonstrated that RES could enhance brown adipocyte formation and thermogenic function and elevate oxygen consumption by activating AMPKα1 (95, 96). Besides, RES dose-dependently decreased triglyceride accumulation in mouse 3T3-L1 preadipocytes via up-regulation of Sirtuin1 (Sirtl) expression (97). Moreover, RES facilitated epinephrine-induced lipolysis, inhibited lipogenesis and glucose conversion to lipids, and counteracted insulin antilipolytic action in rat and human adipocytes (98, 99).

The colonization of HFD-fed obese mice with an “RES-microbiota” (RES-induced gut microbiota) is sufficient to improve lipid metabolism and to ameliorate obesity, suggesting the anti-obesity effects of RES may be partially mediated by the regulation of gut microbiota (100). Due to its poor bioavailability, RES mainly accumulates in the intestinal tract and rarely enters the circulatory system after intake. In the intestinal tract, RES can be metabolized into bioactive small molecules by gut microbiota, which greatly facilitates the regulation of RES on lipid metabolism (100–102). For instance, RES can be biotransformed into 4-hydroxyphenylacetic acid (4-HPA) and 3-hydroxyphenylpropionic acid (3-HPP) by gut microbiota, which conversely improved the gut microbiota composition, as evidenced by the restored abundance of *Lactobacillus, Bifidobacterium*, and *Firmicutes* (103). Thereafter, the improved gut microbiota alleviated obesity through the fasting-induced adipose factor (Fiaf) and sirtuin-1 (Sirt1) signaling pathway (104, 105). On the other hand, RES can lower the level of gut microbial metabolite trimethylamine-N-oxide (a novel risk factor of metabolic syndrome) and enhance bile acid neosynthesis, possibly representing an additional mechanism for the beneficial effects of RES (106). Taken together, these observations raise the possibility that the beneficial effects of RES on obesity are associated with improved gut microbiota (93, 100, 104, 105, 107, 108).

### Grape Seed Proanthocyanidin Extract

Grape seed proanthocyanidin extract (a polymers of flavan-3-ols, GSPE) is the main polyphenolic compound of grape seed extracts, which can transform into anthocyanidin under acid and heating conditions. In addition to its antioxidant function, GSPE also possesses pharmacological effects on cardiovascular disease, inflammatory processes, muscle fatigue, and other metabolic complications (109–111). Recently, increasing evidence has found that GSPE is capable to normalize the disturbance of lipid metabolism and to mitigate obesity.

Works in rodent models found that daily administration of GSPE (250 mg kg^−1^ body weight) to hyperlipidemic rats for 7 days resulted in a 41% reduction in serum TG levels via increasing fecal bile acid and cholesterol excretion (112). In cafeteria diet-induced obese rats, daily delivery of GSPE (25 and 50 mg kg^−1^ body weight) for 21 days resulted in an improvement of mitochondrial function and thermogenic capacity of the BAT, thus increasing energy expenditure and ameliorating obesity (113). Further investigation found that long-term treatment of GSPE (12 weeks) could exert an anti-hyperlipidemia effect, as evidenced by decreased serum levels of TG, TC, and LDL-C and reduced visceral WAT mass (114, 115). Similar results were also obtained in the ovariectomized mice and weaned pigs (116, 117). Consistent with these findings, *in vitro* studies using murine and porcine cell models also elucidated that GSPE could suppress preadipocyte differentiation and proliferation, and promote lipolysis of adipocytes, thus inhibiting adipose cell formation and fat accumulation (118, 119). Besides, both *in vivo* and *in vitro* studies have found that GSPE could prevent low-grade inflammation through inhibiting the production of proinflammatory cytokines [cytokine C-reactive protein (CRP), interleukin-6 (IL-6), and tumor necrosis factor-α] and increasing the production of the anti-inflammatory adipokine adiponectin (120, 121).

Mechanically, GSPE can suppress the disorder of lipid metabolism through regulating gut microbiota. In support, GSPE can restore the obesogenic diet-induced gut microbiota dysbiosis, as manifested by the normalized ratio of *Firmicutes* to *Bacteroidetes* and the increased *Bacteroides* abundance (110, 116, 117). Further evidence comes from the finding that the beneficial effects of GSPE on obesity were abolished when gut microbiota was cleared by antibiotics treatment (110, 117). Interestingly, further investigation suggested that the bacterial metabolites SCFAs (especially propionate) could also communicate in the beneficial effects of GSPE. Alterations of gut microbiota in response to GSPE treatment (250 mg kg^−1^ body weight) in weaned pigs for 28 days led to a 30.2% elevation in the production of propionate compared with the control group (117). On one hand, propionate stimulated the secretion of glucagon like peptide-1 (GLP-1) and peptide YY (PYY, an important regulator of appetite and energy homeostasis) from colonic cells via activation of free fatty acid receptor 2 (FFAR 2), further inhibiting energy intake and fat accumulation (117, 122, 123). On the other hand, propionate could in turn restored the gut microbiota dysbiosis induced by HFD, thus reducing body weight and mitigating obesity (124). In conclusion, these findings suggest that the beneficial effects of GSPE on obesity are partially attributed to the gut microbiota-propionate axis.

### Honokiol

Honokiol (HON) is a natural neolignan derived from the widely used Chinese medicinal herb, *Magnolia officinalis*. HON has been regarded as a promising therapeutic agent for various chronic diseases due to its bioactive effects, such as anti-parkinsonian, anti-inflammatory, anti-cancer, anti-fatigue, and antioxidant properties (125–129). Notably, recent evidence has indicated that HON exerts critical effects on obesity by regulating adipogenesis and lipolysis.

There is evidence showing that HON administration (at doses of 200, 400, and 800 mg kg^−1^ body weight) to diets-induced obese mice for 8 weeks dose-dependently reduced body weight and adipose tissue mass (130). Besides, upon HON treatment (200 mg kg^−1^, for 8 weeks), insulin sensitivity was improved in streptozotocin-induced type 2 diabetic mice by targeting protein tyrosine phosphatase 1B (PTP1B) (131). Apart from using alone, HFD-fed mice fed HON plus magnolol for 16 weeks exhibited a drastically reduction in WAT weight and adipocyte size (132). Consistently, *in vitro* studies using 3T3-L1 adipocytes also demonstrated that HON could exert effects on lipid metabolism, including reducing viability, inducing apoptosis, promoting browning of white adipocytes, improving insulin resistance, and inhibiting apoptosis of brown adipocytes (133–135). Therefore, HON exerts regulatory effects on lipid metabolism and exhibits therapeutical potential against obesity.

Notably, due to its low bioavailability, <10% of HON is absorbed into circulatory system from intestine and the remainders are metabolized by gut microbiota in posterior intestine to generate bioactive molecules and to remodel gut microbiota structure (136). These findings indicate that gut microbiota exerts an irreplaceable role in the lipid metabolic benefits of HON. Using different sexes of HFD-induced obese mice, previous studies have reported that the male mice treated with HON exhibited a significant anti-obesity effect through regulating gut microbiota and metabolites, as manifested by increased *Bacteroides* abundance and reduced lipopolysaccharide (LPS) levels. Nevertheless, HON treatment in female mice did not exhibit the same impact as to male mice (130). In summary, HON plays a vital role in warding off obesity and the related mechanisms might be partially mediated by gut microbiota. However, the detailed mechanisms have not yet been fully identified.

### Capsaicin

**Figure 5:**
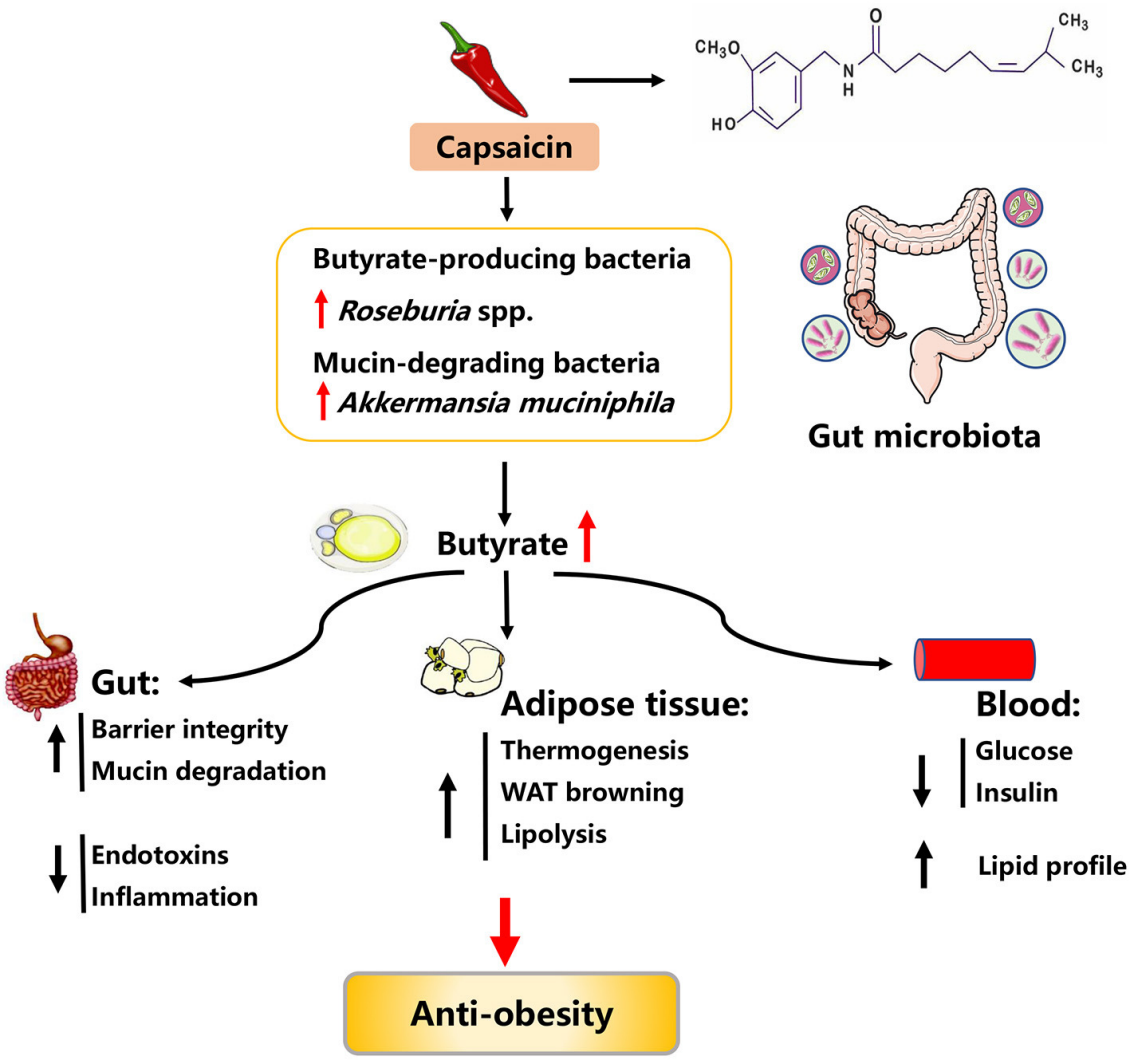
Possible mechanisms explaining the anti-obesity effects of capsaicin.

Capsaicin (CAP) is the major pungent ingredient isolated from red chili peppers (genus *Capsicum*), which are widely consumed as foods and flavoring spices all over the world. CAP has been widely used to treat various diseases because of its bioactive effects, such as anti-cancer, analgesic, neuro-modulating, anti-fatigue, anti-inflammatory properties (137–141). Recently, accumulating evidence has suggested that CAP has beneficial effects on obesity, including reducing lipid accumulation, inducing the browning of WAT, and mitigating inflammation responses (Figure 5).

Using women with gestational diabetes mellitus model, previous studies shown that daily supplementation of 5 mg CAP for 4 weeks can improve serum lipid profiles, as evidenced by lowered levels of fasting serum lipids and postprandial plasma glucose (142). Similar improvements in serum lipid profile were observed in diets-induced obese mice, in which long-term supplementation (5 months) of dietary CAP at a low dose (0.01%) also resulted in a reduction in body weight and adipose tissues mass (143). Apart from the regulation of body weight and serum lipid profile, CAP treatment could improve glucose homeostasis. For instance, treatment of CAP (2 mg kg^−1^ body weight) to transient receptor potential vanilloid-1 (TRPV1)-knockout (KO) obese mice for 12 weeks significantly decreased serum glucose and insulin concentrations (144). Upon further investigation, CAP was found to promote WAT browning and increase thermogenesis (145, 146). Consistently, in 3T3-L1 white adipocytes, a combination treatment of CAP (25 μM) and capsiate (25 μM) could induce browning through activation of peroxisome proliferator-activated receptor γ and β_3_-adrenergic receptor (PPARγ/β_3_-AR) signaling pathway (147). Moreover, CAP time- and dose-dependently inhibited lipid accumulation in 3T3-L1 adipocytes via inducing apoptosis and suppressing adipogenesis (148). Likewise, an *in vitro* study also showed that palmitic acid-treated HepG2 cells exhibited an improvement in lipid metabolism upon CAP treatment (100 μM), as evidenced by decreased lipid accumulation and concentrations of TG and TC as well as increased HDL-C levels (149). In addition, CAP also attenuated obesity-induced inflammatory responses by regulating adipokine release from and macrophage behavior in adipose tissues of obese-mice (150).

Mechanically, CAP exerts anti-obesity effects via altering gut microbiota composition and elevating SCFAs production (Figure 5) (144). In particular, dietary CAP supplementation in diets-induced obese mice could increase the abundance of butyrate-producing bacteria (e.g., *Roseburia* spp.) and promote the generation of butyrate (a metabolic substrate for intestinal epithelial cells), thus improving gut barrier integrity. The improved gut barrier integrity prevented the bacterial endotoxins across the gut barrier and metabolic endotoxemia, thereby ameliorating chronic low-grade inflammation and obesity (151). In line with the alteration in the abundance of butyrate-producing bacteria, the abundance of *Akkermansia muciniphila* (a mucin-degrading bacterium proven to inversely correlate with adiposity) has also been shown to be increased in response to CAP supplementation. However, it remains unclear how dietary CAP supplementation elevated the relative abundance of *Akkermansia muciniphila* (152). Taken together, CAP can serve as a therapeutic agent in the prevention of obesity and the related mechanisms might be associated with gut microbiota-butyrate signaling.

### Konjac Glucomannan

**Figure 6:**
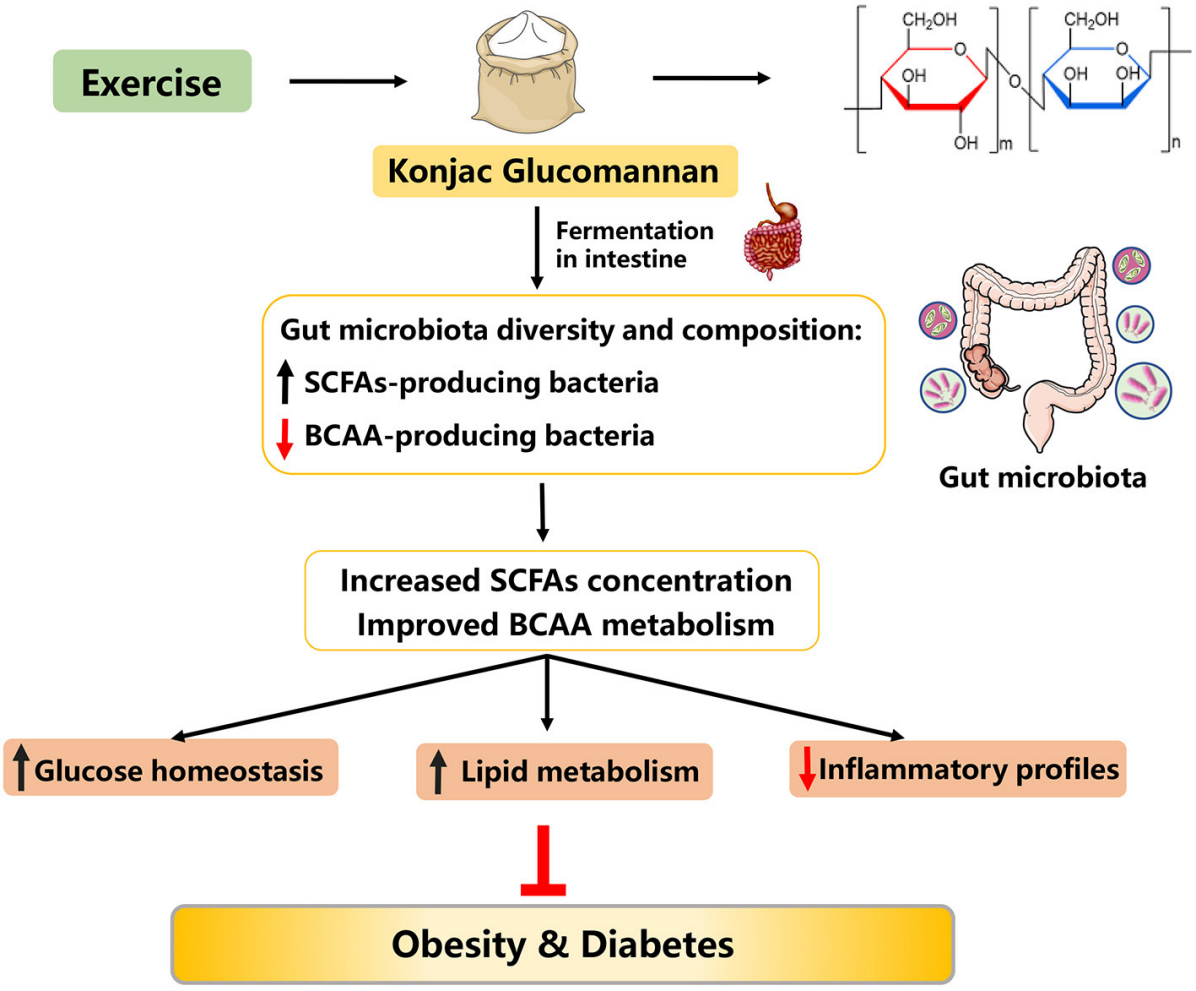
Possible mechanisms explaining the anti-obesity effects of konjac glucomannan.

Konjac flour (KF) is a powder processed from the konjac tuber (*Amorphophallus konjac*), which has traditionally been consumed as a food source and as a component for traditional Chinese medicine in Asian countries for centuries. Konjac glucomannan (KGM), a primary ingredient of KF, is a hydrophilic dietary fiber composed of D-glucose and D-mannose residues connected by β-1,4-glycosidic bonds. It has been widely used as a food additive and dietary supplements in medical practice, pharmaceutical engineering, nutraceutical and food industry (153). KGM has multiple pharmacological effects for the management of diseases such as constipation, wound healing, and colitis (154–156). Recently, growing evidence has suggested that KGM could ameliorate lipid dysmetabolism and exhibit hypoglycemic effects (Figure 6).

It has been shown that dietary KGM supplementation (3.6 g/day) in type 2 diabetic patients with hyperlipidemia for 4 weeks reduced the concentrations of plasma cholesterol, LDL-C, and fasting glucose by 11.1, 20.7, and 23.2%, respectively (157). When combined with exercise, treatment of overweight humans with KGM (3,000 mg/day) for 8 weeks significantly improved blood lipid levels and body composition (158). Similar results were also found in diabetic rodent models (159). In addition, long-term supplementation of dietary KGM (4%, for 16 weeks) to nutritional obese mice effectively decreased body weight, improved lipid metabolism, and inhibited insulin resistance (160, 161). Moreover, in type 2 diabetic rats induced by HFD and streptozotocin, KGM administration (80 mg kg^−1^ body weight, for 28 days) attenuated oxidative stress by regulation of nuclear factor erythroid 2-related factor 2 (Nrf2) pathway and decreased inflammatory responses through regulating nuclear factor-kappa B (NF-κB) pathway, thus ameliorating hyperglycemia (162). Taken together, KGM supplementation, alone or together with exercise, may be a good nutritional tool to prevent and to treat obesity.

KF is a native soluble dietary fiber with high molecular weight, viscosity, and swelling capacity in the intestine, which can remarkably speed up the peristalsis of bowel and slow down the movement of gut microbiota across the cecum (161, 163). Notably, low grain size of KF can swell in intestinal tract more easily and rapidly compared with the native KF (164). Evidence from rodents (161, 165, 166), sows (167), and humans (168) models suggests that alteration of gut microbiota is a contributing mechanism for the anti-obesity effects of KF or/and KGM supplementation. In detail, dietary KF supplementation could normalize the gut microbiota dysbiosis induced by HFD, such as improving the gut microbiota diversity and composition (161, 166). In turn, the amendatory gut microbiota could accelerate the fermentation of dietary KF to improve the SCFAs' concentration in the intestinal contents, thus ameliorating obesity and its-related complications (161, 163). On the other hand, it is well known that increased circulating BCAA concentrations can deteriorate host glucose and lipid metabolism, and subsequently prognosticate future risk of developing insulin resistance and diabetes (169). Interestingly, a recent study has demonstrated that KGM treatment (160 mg kg^−1^) of type 2 diabetic rats for 4 weeks reduced the abundance of BCAA-producing bacteria and improved BCAA metabolism, further ameliorating host lipid metabolism and diabetes (169). In summary, KF might be a promising agent to prevent and to treat obesity, and the potential mechanism was associated with reprogramming gut microbiota and metabolism, especially decreasing BCAA-producing bacteria abundance (Figure 6).

### Chlorophyll

Chlorophyll is an abundant green pigment and non-nutrient compound ubiquitously found in higher plants and other photosynthetic organisms. Chlorophyll has been widely used to prevent various chronic diseases because of its numerous pharmacological effects, such as anti-cancer, anti-inflammatory, and antioxidant properties (170–172). Recently, more and more studies have reported that chlorophyll also plays important roles in ameliorating obesity. A study found that daily consumption of 5 g green-plant membranes containing chlorophyll in overweight subjects for 3 months led to decreases in body weight and serum cholesterol levels (173). Similar results were obtained in HFD-induced obese mice, in which dietary supplementation of a chlorophyll-rich spinach extract (0.18 mg/10 g body weight, for 13 weeks) reduced body weight gain and inflammation as well as improved glucose tolerance (174, 175). Apart from *in vivo* studies, several *in vitro* studies using a 3T3-L1 cell model also discovered that chlorophyll could regulate lipid metabolism, including inhibition of adipocyte proliferation and differentiation, suppression of adipogenesis and lipid accumulation, and activation of browning (176–178).

Evidence from human and rodent models suggests that chlorophyll can modulate the composition and diversity of gut microbiota (179, 180). First, chlorophyll-rich thylakoid treatment of healthy human subjects for 12 weeks elevated the abundance of total bacteria, especially the *Bacteriodes fragilis* group (180). Studies performed in HFD-fed mice also found that chlorophyll increased the relative abundance of *Blautia, Akkermansia*, and *norank_f_Bacteroidales_S24-7_*group and decreased the relative abundance of *Lactococcus* and *Lactobacillus* (174, 175). Moreover, chlorophyllin, an extract isolated from chlorophyll, could alleviate hepatic fibrosis in mice through restoring the gut microbiota, as evidenced by reduced ratio of *Firmicutes*-to-*Bacteroidetes* populations (179). Therefore, chlorophyll, as the most plentiful green pigment in nature, confers beneficial effects on obesity and the mechanisms might be associated with gut microbiota. However, further research is required to examine the specific mechanisms of chlorophyll-gut microbiota-lipid metabolism axis.

### Others

Apart from the above-mentioned plant extracts, several others are also proved to exert roles in mitigating obesity via reprogramming gut microbiota. For instance, dietary supplementation of *Luffa cylindrica* (L.) Roem (2 g kg^−1^ body weight, for 14 weeks) in diets-induced obese mice could ameliorate adiposity and related metabolic complications via increasing the abundance of SCFAs-producing bacteria and the content of SCFAs (181). *Coreopsis tinctoria*, a multifunctional and widely cultivated plant, could improve blood lipid metabolism in hyperlipidemic models by normalizing the disorders of gut microbiota (182). Garcinol supplementation (0.1% or 0.5%) to HFD-fed mice for 13 weeks restored the gut dysbiosis, as evidenced by the augmented *Bacteroidetes*-to-*Firmicutes* ratio, which further dose-dependently improved plasma lipid profile and reduced adipose tissue weight and body weight gain (183). Similarly, diets-induced obese mice treated with a cranberry extract (200 mg kg^−1^, for 8 weeks) exhibited improved insulin resistance and ameliorated obesity through elevating the relative richness of *Akkermansia*, a mucin-degrading bacterium (184). Additionally, *Nitzschia laevis* extract supplementation (10 and 50 mg/kg/day, respectively) for 8 weeks to HFD-fed mice could inhibit lipid accumulation and body weight gain, and the effects were associated with the alteration of gut microbial richness and diversity (185).

## Conclusions

Obesity is one of the most serious public health problems and has increased at an alarming rate worldwide. Currently, various plant extracts, such as mulberry leaf extracts, policosanol, CM, GTE, RES, GSPE, HON, CAP, KGM, and chlorophyll, are demonstrated to regulate serum lipid profile, inflammatory response, browning of WAT, insulin resistance, lipid and glucose metabolism, and other metabolic processes, thus improving obesity and related metabolic disorders (Table 1). A number of experimental studies have elucidated the potential regulatory mechanisms of beneficial effects of plant extracts on obesity and related diseases are closely associated with alterations in host gut microbiota. Hence, plant extracts seem to be promising agents to treat obesity and even other related metabolic syndrome. However, there are still many questions that need to be elucidated: the regulatory efficiency of plant extracts from different origins and conditions; the specific mechanisms of plant extracts targeting gut microbiota; and the anti-obesity effects of plant extracts in different animals and humans under different metabolic conditions. In the near future, more efforts should be made to fully understand the role of plant extracts in host lipid dysmetabolism, thus providing better nutritional strategies to control obesity and to maintain healthy life.

**Table 1:** Summary of the effects of plant extracts on gut microbiota, obesity and its related metabolic disorder.

**Plant extracts**	**Experimental model and subjects**	**Plant extract treatment**	**Microbiota effects**	**Association**	**Response**	**References**
FML	HFD-induced obese mice	FML (240 mg/kg/day) via oral gavage for 6 weeks	*Firmicutes*/*Bacteroidetes* ratio ↓; *Clostridiales* ↑	Acetic acid production ↑	Lipid accumulation, liver steatosis and the whitening of BAT ↓	(28)
Mulberry leaves	HFD-induced obese mice	Mulberry leaves (20%) for 13 weeks	*Firmicutes*/*Bacteroidetes* ratio and *Proteobacteria* ↓; *Akkermansia* ↑	SCFAs production ↑	Body weight gain, fat accumulation and fasting blood glucose ↓; insulin sensitivity ↑	(33)
CM	HFD-induced obese mice	CM (0.4 g crude drug/kg) for 6 weeks	*Bacteroides* and *Akkermansia* ↑; *Mucispirillum* ↓	Microbiota-blood metabolites-liver axis	Adiposis and insulin resistance ↓; glucose uptake ↑	(70)
Paeonol	Alcohol-induced ALD mice	Paeonol (480 mg/kg) for 11 days	*Candida albicans* ↓	mycobiota-mediated dectin-1/IL-1β signaling pathway ↓	ALD inflammatory response and liver fat lesions ↓	(71)
Green tea infusions	HFD-induced obese C57BL/6J mice	Green tea infusions as drinking water for 13 weeks	*Alistipes, Rikenella, Lachnospiraceae, Akkermansia, Bacteroides, Allobaculum* and *Parabacteroides* ↑	SCFAs, gastrointestinal immunity and gut barrier function ↑	Body weight gain, adipose tissue accumulation, hyperglycemia, hypertriglyceridemia, and hypercholesterolemia ↓	(82)
GTE	HFD-induced obese Swiss albino mice	GTE (200 mg/kg) combined with isomalto-oligosaccharide (1 g/kg) for 12 weeks	*Lactobacillus, Bifidobacteria, Akkermansia* and *Roseburia* spp. ↑	Metabolic endotoxemia ↓	Adiposis, lipid accumulation in liver and fasting blood glucose ↓	(83)
GTE	HFD-induced obese C57BL/6J mice	GTE (2%) for 8 weeks	*Firmicutes*/*Bacteroidetes* ratio ↓; *Bifidobacterium* and *Lactobacillus* ↑	Gut barrier function ↑; endotoxin translocation ↓	Adiposis and its related inflammatory response ↓	(84)
Green tea water extracts	HFD-induced obese C57BL/6J mice	Green tea water extracts (1%) for 28 weeks	Family *Rikenellaceae* and *Desulfovibrionaceae* ↓	SCFAs production ↑; endotoxin LPS production ↓	Glucose tolerance ↑; body weight gain, hepatic lipids, and WAT weights ↓	(85)
Green tea polyphenols	HFD-induced obese C57BL/6 mice	Green tea polyphenols (200 mg/kg) for 12 weeks	*Lachnospiraceae, Bacteroides, Alistipes*, and *Faecalibaculum* ↑	the maintaining of intestinal redox state ↑	Lipid metabolism ↑; hyperlipidemia and inflammation ↓	(86)
RES	HFD-induced obese C57BL/6J mice	RES by gavage (300 mg/kg/day) for 16 weeks	*Desulfovibrio, Lachnospiraceae_NK4A316_group* and *Alistipes* ↓; *Allobaculum, Bacteroides* and *Blautia* ↑	Gut intestinal barrier integrity and intestinal redox state ↑	Body weight, liver steatosis and NAFLD ↓; lipid metabolism and insulin resistance ↑	(93, 100, 103)
RES	HFD-induced obese mice	RES (200 mg/kg/day) for 12 weeks	*Lactobacillus* and *Bifidobacterium* ↑; *Firmicutes*/*Bacteroidetes* ratio and *Enterococcus faecalis* ↓	Fiaf signaling pathway ↑	Body and visceral weights, blood glucose, and lipid levels ↓	(104)
RES	HFD-induced obese C57BL/6J mice	RES (0.4%) for 4 weeks	*Lactobacillus* and *Bifidobacterium*↑; *Firmicutes*/*Bacteroidetes* ratio and *Proteobacteria* ↓	Sirtuin-1 signaling pathway ↑	Fat accumulation ↓; WAT browning ↑	(105)
RES	HFHS-induced obese C57Bl/6N mice	RES (0.4%) for 8 weeks	*Turicibacteraceae, Moryella, Lachnospiraceae*, and *Akkermansia* ↓; *Bacteroides* and *Parabacteroides* ↑	Inflammatory state ↓	Glucose homeostasis and insulin sensitivity ↑	(108)
GSPE	HFD-induced obese C57BL/6 mice	GSPE by gavage (300 mg/kg/day) for 7 weeks	*Clostridium* XIVa, *Roseburia*, and *Prevotella* ↑	Inflammatory response ↓	Insulin sensitivity ↑; visceral fat accumulation and adiposity ↓	(110)
GSPE	Weaned pigs at day 28	GSPE (250 mg/kg) for 28 days	*Firmicutes*/*Bacteroidetes* ratio↓; *Akkermansia, Alistipes* and *Bacteroides* ↑	Propionate production ↑	Adipose accumulation and inflammation ↓; lipid metabolism ↑	(117)
HON	HFD-induced obese C57BL/6 mice	HON (200, 400, 800 mg/kg) for 8 weeks	*Akkermansia* and *Bacteroides* ↑; *Oscillospira*↓	SCFAs production ↑; endotoxin LPS production ↓	Body weight, adipose tissue weight, insulin resistance and blood lipid ↓	(130)
CAP	HFD-induced obese C57BL/6J mice	CAP (2 mg/kg) for 12 weeks	*Bacteroides, Coprococcus, Prevotella* and *Akkermansia* ↑; *Proteobacteria* spp. ↓	SCFAs production ↑; inflammatory response ↓	Body weight gain and food intake ↓; lipid profile ↑	(144)
CAP	HFD-induced obese C57BL/6J mice	CAP (0.01%) for 12 weeks	*Ruminococcaceae* and *Lachnospiraceae* (including *Roseburia* spp.) ↑; family *S24_7* ↓	Butyrate production ↑; endotoxin LPS production ↓	Chronic low-grade inflammation, body weight gain and adiposity ↓	(151)
CAP	HFD-induced obese C57BL/6 mice	CAP (0.01%) for 9 weeks	*Proteobacteria* ↓; *Akkermansia muciniphila* ↑	Intestinal mucin degradation ↑	Body weight ↓; glucose tolerance and glucose homeostasis ↑	(152)
KGM	HFD-induced obese C57BL/6J mice	KGM (4%) combined with bacterial cellulose (4%) for 16 weeks	*Firmicutes* and *Mucispirillum* ↓; *Bacteroidetes* and *Akkermansia* ↑	SCFAs production and intestinal integrity ↑	Glucose homeostasis, fatty acid profiles and lipid metabolism ↑; body weight ↓	(161)
Konjaku flour	HFD-induced obese C57BL/6J mice	Konjaku flour (300 mg/day) for 12 weeks	*Megasphaera elsdenii* and *Aerococcaceae* ↑; *Alistipes, Alloprevotella* and *Bacteroides acidifaciens* ↓	Gut Intestinal barrier function ↑	Body weight gain, fat mass and inflammatory state ↓	(166)
KGM	streptozotocin-induced type 2 diabetic rats	KGM (160 mg/kg) for 4 weeks	*Clostridium* spp., *Bacteroides* spp., *Prevotella* spp., *Klebsiella* spp., *Streptococcus* spp., and *S*. *aureus* ↓	Bacterial-associated BCAAs ↓; BCAA metabolism ↑	Type 2 diabetes ↓; lipid and glucose metabolism ↑	(169)
Chlorophyll	HFD-induced obese C57BL/6J mice	Chlorophyll-rich spinach extract (0.18 mg/10 g /day) for 13 weeks	*Blautia, norank_f_Bacteroidales_S24-7_group* and *Akkermansia* ↑; *Lactobacillus* and *Lactococcus* ↓	SCFAs production ↑; endotoxin LPS production ↓	Body weight gain and low-grade inflammation ↓; glucose tolerance ↑	(174, 175)
Chlorophyllin	Carbon tetrachloride-induced liver fibrosis BALB/c mice	Chlorophyllin (5 mg/kg) for 6 weeks	*Firmicutes*/*Bacteroidetes* ratio ↓	Plasma endotoxin concentration ↓	Hepatic inflammation and liver fibrosis ↓	(179)

*FML, flavonoids from mulberry leaves; HFD, high fat diets; BAT, brown adipose tissue; SCFAs, short chain fatty acids; CM, cortex moutan; ALD, alcoholic liver disease; IL-1β, interleukin-1β; GTE, green tea extract; LPS, lipopolysaccharide; NAFLD; nonalcoholic fatty liver disease; RES, resveratrol; Fiaf, fasting-induced adipose factor; WAT, white adipose tissue; HFHS, high fat/high sugar; GSPE, grape seed proanthocyanidin extract; HON, honokiol; CAP, capsaicin; KGM, konjac glucomannan; BCAAs, branched-chain amino acids; ↑, promotion; ↓, inhibition*.

## Author Contributions

YD, YY, and JD provided the topic of the review. GW, YZ, BS, JZ, and SZ prepared the manuscript and figures. YD revised the manuscript. All authors approved the final manuscript.

## Funding

This study was jointly supported by the National Natural Science Foundation of China (U19A2037, 31872985, 31802077), the services of alternative antibiotic feed and breeding technology (H20551), the Changsha Natural Science Funds for Distinguished Young Scholar (kq2009020), the Natural Science Foundation of Guangxi Province (2020JJA130102, 2018JJB130239), Special funds for the construction of innovative provinces in Hunan Project (2019RS3022), China Agriculture Research System of MOF and MARA (CARS-35), the Strategic Priority Research Program of the Chinese Academy of Sciences (XDA24030204), and Open Fund of Key Laboratory of Agro-ecological Processes in Subtropical Region, Chinese Academy of Sciences (ISA2020203).

## Conflict of Interest

The authors declare that the research was conducted in the absence of any commercial or financial relationships that could be construed as a potential conflict of interest.

## Publisher's Note

All claims expressed in this article are solely those of the authors and do not necessarily represent those of their affiliated organizations, or those of the publisher, the editors and the reviewers. Any product that may be evaluated in this article, or claim that may be made by its manufacturer, is not guaranteed or endorsed by the publisher.
